# A Predicted Model for Refractory/Recurrent Cytomegalovirus Infection in Acute Leukemia Patients After Haploidentical Hematopoietic Stem Cell Transplantation

**DOI:** 10.3389/fcimb.2022.862526

**Published:** 2022-03-22

**Authors:** Meng-Zhu Shen, Shen-Da Hong, Jie Wang, Xiao-Hui Zhang, Lan-Ping Xu, Yu Wang, Chen-Hua Yan, Huan Chen, Yu-Hong Chen, Wei Han, Feng-Rong Wang, Jing-Zhi Wang, Kai-Yan Liu, Xiao-Jun Huang, Xiao-Dong Mo

**Affiliations:** ^1^ Peking University People’s Hospital, Peking University Institute of Hematology, National Clinical Research Center for Hematologic Disease, Beijing Key Laboratory of Hematopoietic Stem Cell Transplantation, Beijing, China; ^2^ National Institute of Health Data Science at Peking University, Peking University Health Science Center, Beijing, China; ^3^ Department of Hematology, The Second Affiliated Hospital of Shandong First Medical University, Shandong, China; ^4^ Peking-Tsinghua Center for Life Sciences, Academy for Advanced Interdisciplinary Studies, Peking University, Beijing, China; ^5^ Research Unit of Key Technique for Diagnosis and Treatments of Hematologic Malignancies, Chinese Academy of Medical Sciences, Beijing, China

**Keywords:** cytomegalovirus, haploidentical donor, hematopoietic stem cell transplant, predicted model, refractory

## Abstract

**Objective:**

We aimed to establish a model that can predict refractory/recurrent cytomegalovirus (CMV) infection after haploidentical donor (HID) hematopoietic stem cell transplantation (HSCT).

**Methods:**

Consecutive acute leukemia patients receiving HID HSCT were enrolled (n = 289). We randomly selected 60% of the entire population (n = 170) as the training cohort, and the remaining 40% comprised the validation cohort (n = 119). Patients were treated according to the protocol registered at https://clinicaltrials.gov (NCT03756675).

**Results:**

The model was as follows: Y = 0.0322 × (age) – 0.0696 × (gender) + 0.5492 × (underlying disease) + 0.0963 × (the cumulative dose of prednisone during pre-engraftment phase) – 0.0771 × (CD34+ cell counts in graft) – 1.2926. The threshold of probability was 0.5243, which helped to separate patients into high- and low-risk groups. In the low- and high-risk groups, the 100-day cumulative incidence of refractory/recurrent CMV was 42.0% [95% confidence interval (CI), 34.7%–49.4%] vs. 63.7% (95% CI, 54.8%–72.6%) (*P* < 0.001) for total patients and was 50.5% (95% confidence interval (CI), 40.9%–60.1%) vs. 71.0% (95% CI, 59.5%–82.4%) (*P* = 0.024) for those with acute graft-versus-host disease. It could also predict posttransplant mortality and survival.

**Conclusion:**

We established a comprehensive model that could predict the refractory/recurrent CMV infection after HID HSCT.

**Clinical Trial Registration:**

https://clinicaltrials.gov, identifier NCT03756675.

## 1 Introduction

Allogeneic hematopoietic stem cell transplantation (allo-HSCT) is the most important curative therapy for patients with acute leukemia (AL) (Xu et al., 2018; Zhang et al., 2021). Human leukocyte antigen (HLA)-haploidentical related donors (HIDs) have become important donors. Such donors account for 60% of all allo-HSCT donors in China (Xu et al., 2021) and account for 42% of allo-HSCT family donors in Europe (Passweg et al., 2021).

Infection is a major cause of transplant-related mortality after HID HSCT (Yan et al., 2016). Cytomegalovirus (CMV) infection is the most common infection after HID HSCT, with a cumulative incidence of approximately 60%–70% in patients receiving an antithymocyte globulin (ATG)-based regimen (Chen et al., 2016; Mo et al., 2016) and 40%–50% in those receiving a posttransplant cyclophosphamide (PTCy) regimen (Crocchiolo et al., 2016; Goldsmith et al., 2016). Preemptive antiviral therapies can prevent the development of CMV infection; however, side effects (e.g., myelosuppression with ganciclovir and nephrotoxicity with foscarnet sodium) are inevitable and may be magnified due to poor graft function (PGF). Additionally, conditioning regimen-related toxicities are more common in HID HSCT recipients (Lu et al., 2006; Sun et al., 2015). In addition, 50.6% and 30.3% of these patients show respective refractory and recurrent CMV infections, despite the use of anti-CMV therapies. Refractory/recurrent CMV infection is an independent risk factor for non-relapse mortality (NRM) (Liu et al., 2015).

Refractory/recurrent CMV infection may be due to CMV antiviral drug resistance and some CMV-related variables (e.g., previous or prolonged anti-CMV drug exposure) (Chemaly et al., 2019). Some clinical factors, including young age, mismatched family donor transplants, cord blood transplantation, T-cell depletion, use of alemtuzumab and ATG, and acute graft-versus-host disease (aGVHD) may also contribute to refractory/recurrent CMV infection (Miller et al., 1986; Martino et al., 2001; Martin et al., 2006; Park et al., 2009; Ljungman et al., 2011; Chemaly et al., 2019). In addition, Guiu et al. (2020) reported that refractory CMV infection after allo-HSCT only corresponded to a 21.4% rate of resistant virus infection, and most of the anti-CMV therapy failures might be due to clinical resistance. However, there is no comprehensive model for predicting refractory/recurrent CMV infection after HID HSCT.

Most CMV infections occur during the early post-engraftment phase after HID HSCT (the median duration from HID HSCT to CMV infection is 31–35 days) (Liu et al., 2015; Chen et al., 2016). CMV becomes latent in non-dividing cells, and reconstitution of blood elements post-HSCT stimulates the replication of CMV and provides a milieu for CMV reactivation (Blume and Thomas, 2016). Thus, patients with a higher risk of CMV infection may need to receive further prophylaxis as soon as neutrophil engraftment is achieved, and variables in the pre-engraftment phase are critical for creating the predicted model.

In the present study, we propose a comprehensive model for predicting refractory/recurrent CMV infection after HID HSCT based on patient characteristics and pre-engraftment variables (i.e., donor/recipient CMV serological status and corticosteroid exposure).

## 2 Patients and Methods

### 2.1 Study Design

Consecutive AL patients receiving HID HSCT between March 5, 2020, and January 1, 2021, at Peking University, Institute of Hematology (PUIH), were enrolled. The end point of the last follow-up for all survivors was November 11, 2021. All patients were treated according to the protocol registered at https://clinicaltrials.gov (NCT03756675). The study was conducted in accordance with the *Declaration of Helsinki*, and the protocol was approved by the institutional review board of Peking University People’s Hospital. Informed consent was obtained from all patients or their guardians.

### 2.2 Transplant Regimens

Major conditioning regimens consisted of cytarabine, busulfan, cyclophosphamide, and semustine (Wang et al., 2015; Mo et al., 2018; Wang et al., 2021). Granulocyte colony-stimulating factor-primed peripheral blood (PB) harvests were administered to the recipients on the same day of collection. ATG, cyclosporine A (CSA), mycophenolate mofetil, and short-term methotrexate were administered to prevent GVHD (**Supplementary Methods**) (Wang et al., 2019). Protocol for GVHD and minimal residual disease therapy had been reported in detail (Liu et al., 2020; Zhao et al., 2021; Fan et al., 2021; Shen et al., 2021; Mo et al., 2022; Shen et al., 2022; Shen et al., 2022).

### 2.3 Protocol for Cytomegalovirus Monitoring and Therapy

The detailed information for infection prophylaxis and monitoring other than CMV is shown in **Supplementary Methods** (Tomblyn et al., 2009; Mo et al., 2016; Hu et al., 2019).

Ganciclovir (5 mg·kg^-1^) was administered intravenously twice daily for CMV prophylaxis from days –9 to –2. However, considering the side effects of ganciclovir prophylaxis that may negatively impact engraftment, we used acyclovir after donor stem cell infusion instead of ganciclovir (Zaia, 2016). Acyclovir (200–400 mg) was orally administered against the herpes virus. It was administered twice daily from day +1 until 1 year after HSCT or until the time CSA was discontinued in those who received CSA for more than 1 year after HSCT. Quantitative polymerase chain reaction (PCR) analysis for plasma CMV copies was conducted at least weekly until day +100. For the patients who received systemic immunosuppressive therapies after day +100 [e.g., receiving ruxolitinib for chronic GVHD (cGVHD)], they should also monitor the plasma CMV copies regularly.

Preemptive antiviral therapy with either intravenous ganciclovir or foscarnet was given when the PCR tests were positive for >1 × 10^3^ copies/ml CMV in a single test on PB. For the patients receiving preemptive therapy, PCR analysis for plasma CMV copies was performed at least twice weekly. Preemptive therapy was continued until 2 negative assays were obtained. The combination of ganciclovir and foscarnet could be considered for patients who showed refractory/recurrent CMV infections (Ljungman et al., 2019). The diagnosis and therapies for CMV disease were according to the international criteria (Ljungman et al., 2017; Ljungman et al., 2019).

### 2.4 Building Machine Learning Models

**Figure 1:**
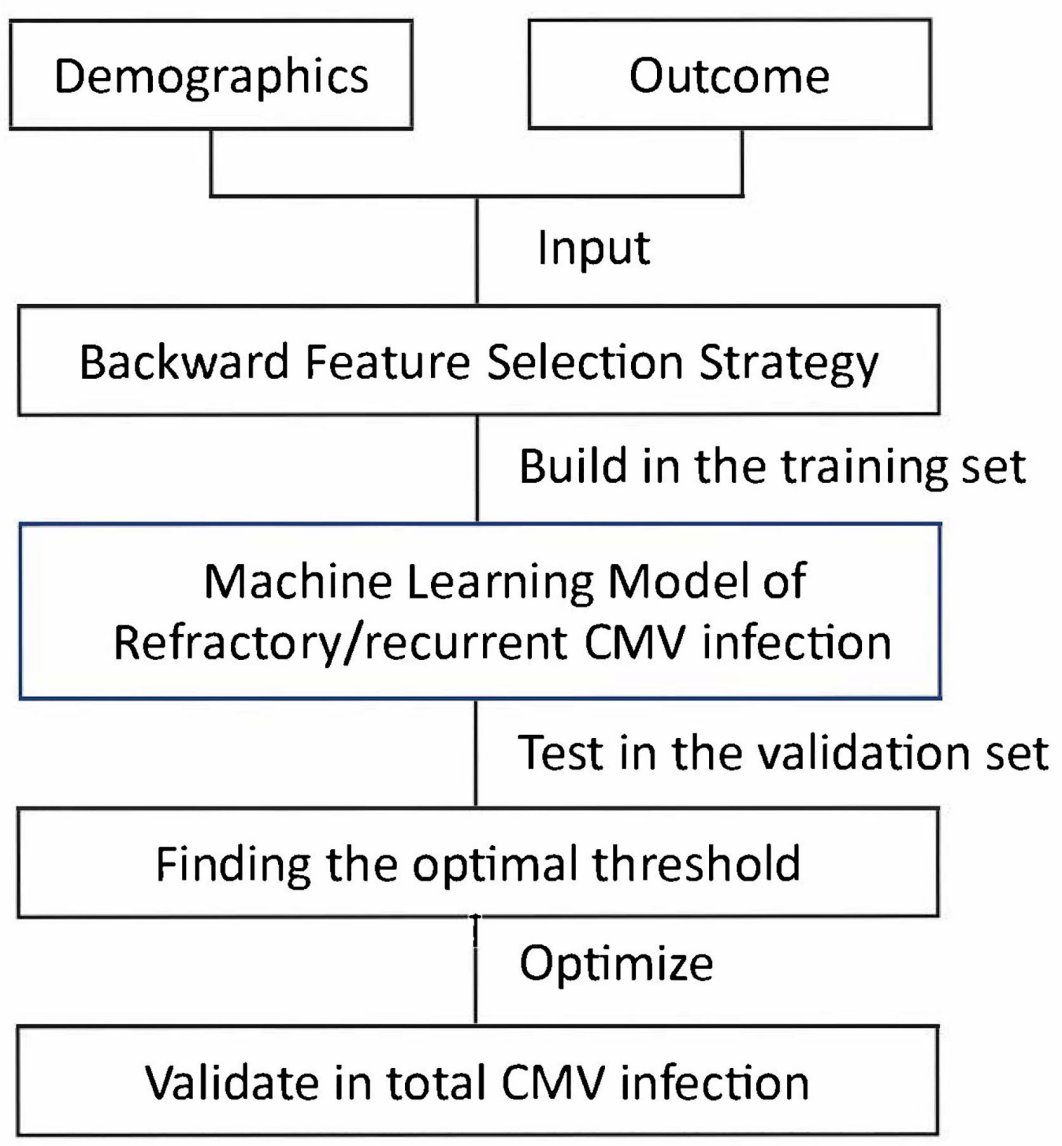
Flow diagram of building the machine learning model.

Our method consisted of three steps: selecting prognostic variables, building models, and finding the optimal threshold (**Figure 1**) (Nelder and Wedderburn, 1972; Hosmer and Lemeshow, 1989; Zweig and Campbell, 1993; Guyon and Andre, 2003; Hastie, 2009; Seabold and Perktold, 2010; Guo et al., 2016).

#### 2.4.1 Backward Feature Selection Strategy

We randomly selected 60% of the entire population (n = 170) as the training cohort, and the remaining 40% comprised the validation cohort (n = 119). For primary outcome (i.e., refractory/recurrent CMV infection), the model-building steps were performed in the training cohort and verified in the validation cohort. The sensitivity, specificity, area under the curve (AUC) score, and accuracy score were identified in both the training and validation cohorts.

We used feature selection techniques to select the predictive variables (**Supplementary Methods**) (Guyon and Andre, 2003). By doing this, we could reduce the complexity of the machine learning model while improving the generalizability. We set age and gender to be obligate variables in the machine learning model. For other variables, we selected the top 3 significant variables using backward feature selection strategy. In detail, we started with all variables including age and gender. At each iteration, we removed the least significant variable (variable with the highest *P* value) except age and gender. Aside from the involved variables, we also added an extra constant variate to make the feature selection more robust. The selection was realized using generalized linear models with binomial exponential family distribution of statsmodels v0.13.0 statistical models module with Python 3.8 based on anaconda3 development platform (Nelder and Wedderburn, 1972).

#### 2.4.2 Building Models

We used generalized linear models with binomial exponential family distribution to realize logistic regression models, which were equivalent models. Aside from the selected variables, we added an extra constant variate for the predicted model to make the machine learning models stronger. We used statsmodels v0.13.0 with Python 3.8 to build the models based on anaconda3 development platform. The model parameters were set to be the defaults (Hosmer and Lemeshow, 1989; Hastie, 2009; Seabold and Perktold, 2010).

#### 2.4.3 Finding the Optimal Threshold

Logistic regression model produced values between 0 and 1, which could be treated as the probabilities to be positive prediction. We needed to determine the threshold of output positive predictions (1) or negative predictions (0). In detail, we drew receiver operating characteristic (ROC) curves (Zweig and Campbell, 1993) and calculated the g-mean for each threshold (Guo et al., 2016). The best threshold corresponded to the largest g-mean. The g-mean was calculated as sqrt (tpr×(1-fpr)), where tpr represented true positive rate, fpr represented false positive rate, under a given threshold.

#### 2.4.4 Evaluation for Model

ROC-AUC was defined as the AUC of the true positive rate vs. the false positive rate at various thresholds ranging from 0 to 1. Confusion matrix was a summary table of predictions. In this paper, the confusion matrix was of 2 × 2 shape. The diagonal showed the count values of correct predictions, while the others showed the count values of incorrect predictions. Besides, we also normalized the count values by the number of True Label (Outcome) or the number of Predicted Label (Prediction). To better visualize the matrix, we colored the values with Blues colorbar.

### 2.5 Definitions

The diagnosis for CMV infections was according to the international criteria (Ljungman et al., 2017; Chemaly et al., 2019). Particularly, the refractory/recurrent CMV infection included: 1) CMV DNA levels increasing (e.g., more than 1 log_10_); 2) CMV viral load continuing at the same level; 3) worsening in signs/symptoms or progression into end-organ disease; or 4) lack of improvement in signs/symptoms after at least 2 weeks of appropriately dosed anti-CMV therapy. The diagnosis of aGVHD (Harris et al., 2016) and cGVHD (Jagasia et al., 2015) was made according to international criteria.

The definitions for Disease Risk Index (DRI), engraftment, PGF (Sun et al., 2015), relapse, NRM, leukemia-free survival (LFS), and overall survival (OS) are shown in **Supplementary Methods** (Mo et al., 2021).

### 2.6 Statistical Methods

In the present study, the primary outcome was refractory/recurrent CMV infection. The secondary outcomes included total CMV infection, relapse, NRM, LFS, and OS.

Mann–Whitney *U*-test was used to compare continuous variables; χ^2^ and Fisher’s exact tests were used for categorical variables. The Kaplan–Meier method was used to estimate the probability of LFS and OS. Competing risk analyses were performed to calculate the cumulative incidence of CMV infection, relapse, and NRM (Gooley et al., 1999). Testing was two-sided at the *P* < 0.05 level. Statistical analysis was performed on SPSS 22.0 software (SPSS, Chicago, IL) and R software (version 4.0.0) (http://www.r-project.org).

## 3 Results

### 3.1 Patient Characteristics

A total of 289 patients were enrolled, and the characteristics were all comparable between training and validation cohorts (**Table 1**). All patients achieved neutrophil engraftment, and the median time from HSCT to neutrophil engraftment was 12 days (range, 9–28 days). Two hundred seventy-nine (96.5%) patients achieved platelet engraftment, and the median time from HSCT to platelet engraftment was 13 days (range, 7–144 days), respectively.

**Table 1:** Patient characteristics.

Characteristics	Training cohort (*n* = 170)	Validation cohort (*n* = 119)	*P* value
Median age at allo-HSCT, years (range)	29 (1–66)	30 (1–64)	0.754
Gender, *n* (%)			0.153
Male	42 (24.7)	21 (17.6)	
Female	128 (75.3)	98 (82.4)	
Underlying disease, *n* (%)			0.481
Acute myeloid leukemia	90 (52.9)	68 (57.1)	
Acute lymphoblastic leukemia	80 (47.1)	51 (42.9)	
Disease status before allo-HSCT, *n* (%)			0.057
CR1	122 (71.8)	97 (81.5)	
>CR1	48 (28.2)	22 (18.5)	
Disease Risk Index before allo-HSCT, *n* (%)			0.368
Low risk	7 (4.1)	6 (5.0)	
Intermediate risk	126 (74.1)	92 (77.3)	
High risk	37 (21.8)	21 (17.6)	
HCT-CI scores before allo-HSCT, *n* (%)			0.602
0 (low risk)	136 (80.0)	91 (76.5)	
1–2 (intermediate risk)	24 (14.1)	26 (21.9)	
≥3 (high risk)	10 (5.9)	2 (1.6)	
Number of HLA-A, HLA-B, HLA-DR mismatches, *n* (%)			0.658
1 locus	3 (1.8)	3 (2.5)	
≥2 loci	167 (98.2)	116 (97.5)	
Cytomegalovirus serostatus before HSCT, *n* (%)			0.848
Donor+/recipient-	5 (2.9)	4 (3.4)	
Donor+/recipient+	159 (93.5)	111 (93.3)	
Donor-/recipient+	6 (3.5)	4 (3.4)	
Conditioning regimen, *n* (%)			0.509
Chemotherapy-based regimen	167 (98.2)	118 (99.2)	
TBI-based regimen	3 (1.8)	1 (0.8)	
Median cumulative dose of prednisone during pre-engraftment phase, (mg/kg)	3.63 (0.73–12.82)	3.55 (0.72–16.94)	0.726
Donor/recipient gender matched, *n* (%)			0.703
Female donor/male recipient combination	34 (20.0)	26 (21.8)	
Others	136 (80.0)	93 (78.2)	
Donor/recipient relation, *n* (%)			0.662
Mother donor	20 (11.8)	9 (7.6)	
Collateral donor	2 (1.2)	4 (3.4)	
Others	148 (87.1)	106 (89.1)	
MNC counts in graft, median (range, ×10^8^/kg)	9.41 (5.20–16.02)	9.04 (5.41–27.52)	0.547
CD34+ cell counts in graft, median (range, ×10^6^/kg)	3.39 (0.75–14.29)	3.71 (1.10–29.35)	0.609
Median follow-up of survivors, days (range)	258 (66–490)	279.5 (52–409)	0.520

allo-HSCT, allogeneic hematopoietic stem cell transplantation; CR, complete remission; HLA, human leukocyte antigen; HCT CI, hematopoietic cell transplantation-specific comorbidity index; MNC, mononuclear cell; TBI, total body irradiation.

The 100-day cumulative incidence of aGVHD after HID HSCT was 57.8% [95% confidence interval (CI), 52.1%–63.5%]. The 1-year cumulative incidence of cGVHD after HID HSCT was 30.5% (95% CI, 24.6%–36.4%). The 1-year cumulative incidence of PGF after HID HSCT was 3.1% (95% CI, 1.1%–5.1%). Twenty-nine (10.0%) patients experienced relapse, and 11 (3.8%) patients died of NRM. Two hundred seventy-five (95.2%) patients survived until the last follow-up, and the median duration of follow-up was 267 days (range, 52–490 days). The 1-year probabilities of relapse, NRM, LFS, and OS after HID HSCT were 14.6% (95% CI, 8.9%–20.3%), 4.8% (95% CI, 1.8%–7.8%), 80.6% (95% CI, 74.5%–87.1%), and 93.0% (95%CI, 88.9%–97.3%), respectively.

### 3.2 Cytomegalovirus Characteristics

Two hundred thirty-one (79.9%) patients developed CMV infection, and 136 (47.1%) and 29 (10.0%) showed refractory and recurrent CMV infection, respectively. A total of 146 (50.5%) patients showed refractory/recurrent CMV infection (i.e., 19 patients had both refractory and recurrent CMV infection). The median time from HSCT to the first CMV infectious event was 32 days (range, 17–76 days). Five patients experienced CMV disease (pneumonia: 2; enteritis: 2; cystitis: 1). The initial plasma level of CMV DNAemia was 2.4 (range, 1.0–100.0) × 10^3^ copies/ml, and the highest plasma level of CMV DNAemia was 7.23 (range, 1.01–730.00) × 10^3^ copies/ml. The 100-day cumulative incidence of total, refractory, recurrent, and refractory/recurrent CMV infection after HID HSCT was 79.9% (95% CI, 75.3%–84.5%), 47.1% (95% CI, 41.3%–52.9%), 9.7% (95% CI, 6.3%–13.1%), and 50.5% (95% CI, 44.7%–56.3%), respectively.

### 3.3 Predicted Model for Refractory/Recurrent Cytomegalovirus Infection

A predictive model for refractory/recurrent CMV infection was developed (**Figure 1**, **Supplementary Methods**, **Supplementary Table S1**, and **Supplementary Figure S1**), and the equation was as follows:


$$\text{Probability }(\text{refractory/recurrent CMV infection})=\frac{1}{1+\exp(-\text{Y})}$$

where Y = 0.0322 × (age) – 0.0696 × (gender) + 0.5492 × (underlying disease) + 0.0963 × (the cumulative dose of prednisone during pre-engraftment phase) – 0.0771 × (CD34+ cells count in graft) – 1.2926. Particularly, underlying disease included acute myeloid leukemia (value = 0) and acute lymphoblastic leukemia (value = 1). Gender included male (value = 0) and female (value = 1). The age (years), cumulative dose during pre-engraftment phase (mg/kg), and CD34+ cell count (×10^6^/kg) in graft used actual numerical value (**Supplementary Table S1**). Particularly, total corticosteroid dose use during pre-engraftment phase was converted into an equivalent amount of prednisone. The threshold of probability was 0.5243, and the g-mean was 0.635. Patients were separated into low- and high-risk groups by the threshold.

**Figure 2:**
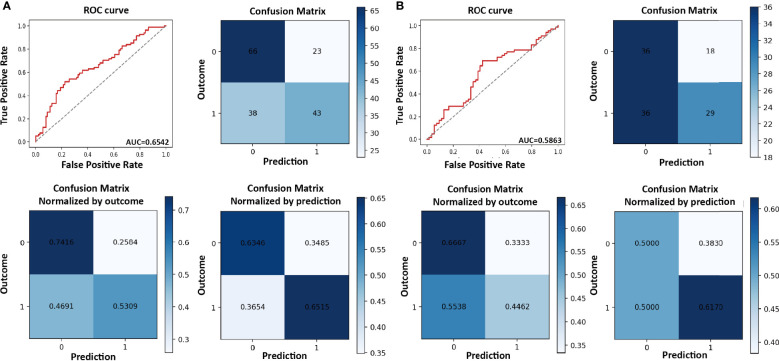
Receiver operating characteristic (ROC) curve and confusion matrix for refractory/recurrent cytomegalovirus (CMV) infection model in the training **(A)** and validation cohorts **(B)**.

In the training cohort, the sensitivity, specificity, AUC score, and accuracy score were 0.531, 0.742, 0.654, and 0.641, respectively. ROC curve for the model and confusion matrix is shown in **Figure 2A** and **Supplementary Table S2**. In the validation cohort, the sensitivity, specificity, AUC score, and accuracy score were 0.446, 0.667, 0.586, and 0.546, respectively. ROC curve for the model and confusion matrix is shown in **Figure 2B** and **Supplementary Table S3**.

### 3.4 Predicted Value of This Model in the Total Population

#### 3.4.1 Refractory/Recurrent Cytomegalovirus Infection

**Figure 3:**
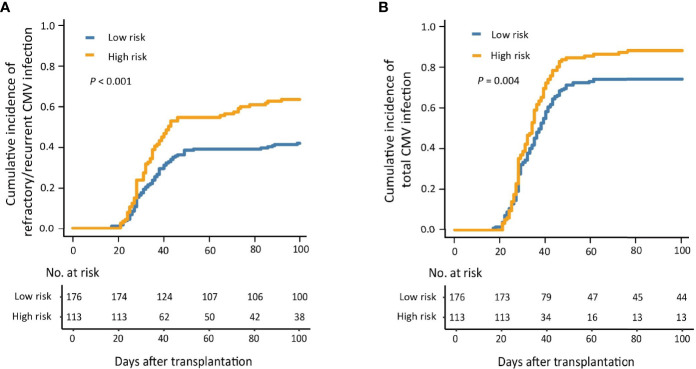
The 100-day cumulative incidence of refractory/recurrent **(A)** and total **(B)** cytomegalovirus (CMV) infection in the low- and high-risk groups.

The 100-day cumulative incidence of refractory/recurrent CMV infection in the low- and high-risk groups was 42.0% (95% CI, 34.7%–49.4%) and 63.7% (95% CI, 54.8%–72.6%), respectively (*P* < 0.001; **Figure 3A**).

For the patients with HCT-CI scores of 0, the 100-day cumulative incidence of refractory/recurrent CMV infection was significantly higher in the high-risk group compared with those in the low-risk group (**Supplementary Figure S2**), which was similar in the patients with HCT-CI scores of ≥1 (**Supplementary Figure S3**).

For the patients in CR1 before HSCT, the 100-day cumulative incidence of refractory/recurrent CMV infection was significantly higher in the high-risk group compared with those in the low-risk group (**Supplementary Figure S4**), which was similar for those in >CR1 before HSCT (**Supplementary Figure S5**). Particularly, for the patients who were in non-remission before HSCT, the 100-day cumulative incidence of refractory/recurrent CMV infection seemed to be higher than that of the low-risk group, although it did not reach statistical significance.

#### 3.4.2 Total Cytomegalovirus Infection

The 100-day cumulative incidence of total CMV infection in the low- and high-risk group was 74.4% (95% CI, 67.9%–80.9%) and 88.5% (95% CI, 82.6%–94.4%), respectively (*P* = 0.004; **Figure 3B**).

#### 3.4.3 Cytomegalovirus Disease

The 100-day cumulative incidence of total CMV disease in the low- and high-risk group was 0.6% (0.0%–1.7%) and 3.5% (0.1%-6.9%), respectively (*P* = 0.059).

### 3.5 Predicted Value of This Model in Patients With and Without aGVHD

**Figure 4:**
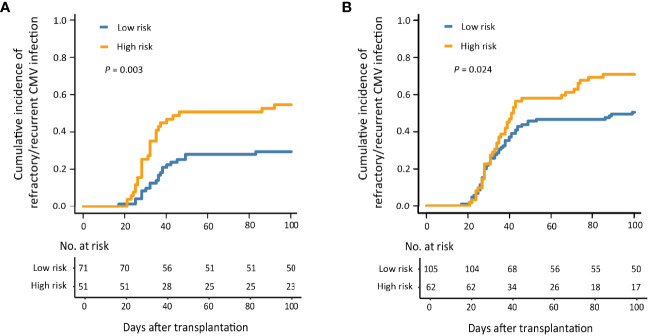
The 100-day cumulative incidence of refractory/recurrent cytomegalovirus (CMV) infection in patients without acute graft-versus-host disease (aGVHD) **(A)** and with aGVHD **(B)**.

In patients without aGVHD (n = 122), the cumulative incidence of refractory/recurrent CMV infection at 100 days after HID HSCT was 29.6% (95% CI, 18.9%–40.3%) and 54.9% (95% CI, 41.0%–68.8%) (*P* = 0.003) in the low- and high-risk groups (**Figure 4A**). In addition, the patients in the low-risk group showed a lower incidence of total CMV infection than that of those in the high-risk group [69.0% (95% CI, 58.1%–79.9%) and 88.2% (95% CI, 79.0%–97.4%), *P* = 0.001].

In patients with aGVHD (n = 167), the cumulative incidence of refractory/recurrent CMV infection at 100 days after HID HSCT was 50.5% (95% CI, 40.9%–60.1%) and 71.0% (95% CI, 59.5%–82.4%), respectively, in the low- and high-risk groups (*P* = 0.024; **Figure 4B**).

### 3.6 Other Clinical Outcomes After Haploidentical Donor Hematopoietic Stem Cell Transplantation

The 100-day cumulative incidence of aGVHD after HID HSCT was comparable between low- and high-risk groups (**Supplementary Figure S6**). The 1-year cumulative incidence of cGVHD after HID HSCT was also comparable between the low- and high-risk groups (**Supplementary Figure S7**).

**Figure 5:**
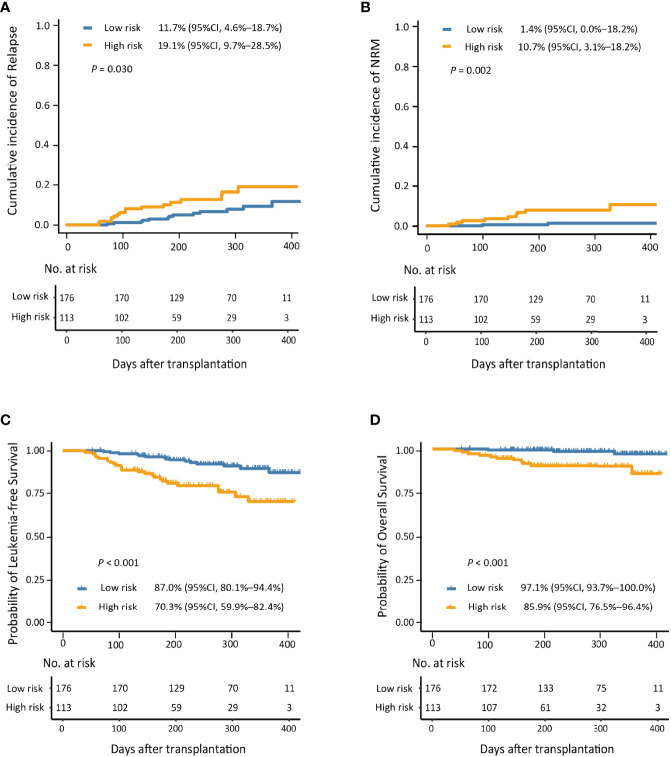
The 1-year cumulative incidence of relapse **(A)**, non-relapse mortality (NRM; **B**), leukemia-free survival (LFS; **C**), and overall survival (OS; **D**) in the low- and high-risk groups.

The 1-year cumulative incidence of PGF after HID HSCT for low-risk group was significantly lower than that of the high-risk group (**Supplementary Figure S8**). In addition, the 1-year cumulative incidence of relapse, NRM, LFS, and OS after HID HSCT of patients in the low-risk group was significantly better compared with those in the high-risk group (**Figure 5**).

## 4 Discussion

In the present study, we propose a predictive model that includes age, sex, underlying disease, CD34+ cell count in the graft, and cumulative dose of corticosteroids during the pre-engraftment phase for refractory/recurrent CMV infection after HID HSCT for the prediction of total CMV infection, mortality, and survival after HID HSCT. To the best of our knowledge, we are the first to integrate different prognostic variables during the pre-engraftment phase and establish a comprehensive model that could effectively predict refractory/recurrent CMV infection in HID HSCT recipients.

The cumulative corticosteroid dose during the pre-engraftment phase was included in the prediction model. Some guidelines recommend that every attempt should be made to reduce or stop immunosuppression, especially corticosteroids, during the treatment of CMV infection after allo-HSCT (Emery et al., 2013). In addition, Nichols et al. (2001) reported that high-dose corticosteroid use (≥2 mg/kg/day) was the only factor significantly associated with increasing CMV antigenemia levels in patients receiving antiviral treatment. In the present study, we first reported a positive association between the cumulative dose of corticosteroids and refractory/recurrent CMV infection after HID HSCT. This might be because high-dose corticosteroids interfere with CMV-directed cytotoxic T lymphocyte (CTL) function (Ljungman et al., 2019). Thus, although corticosteroids are important during the conditioning regimen, which could relieve the infusion reaction of cytarabine, reduce the mucosal edema of the gastrointestinal tract caused by chemotherapies, and prevent serum sickness of ATG (Red Blood Cell Disease (Anemia) Group et al. 2017), their dosage should be controlled during the pre-engraftment phase. On the other hand, corticosteroids are widely used in chemotherapies for patients with ALL, which suggests that these patients would experience higher steroid exposure. This may also increase the risk of refractory/recurrent CMV infection in ALL patients after HID HSCT.

In addition, the CD34+ cell count in the graft was also included in the model. Several studies have observed that a higher CD34+ cell dose in harvests could improve neutrophil and platelet engraftment (Teofili et al., 2020), induce faster monocyte (Bittencourt et al., 2002) and lymphocyte recovery (Patel et al., 2019), and decrease the incidence of fungal infections (Bittencourt et al., 2002). In addition, Arcuri et al. (2020) reported that, in patients receiving alternative donor allo-HSCT, the incidence of CMV infection was lower in those who received higher doses of CD34+ cells.

With the help of our models, we identified high-risk patients who would have refractory/recurrent CMV infection at an early stage after HID HSCT. Because there is an estimated 3-week window of opportunity between neutrophil engraftment (median, +12 days after HSCT) and the first episode of CMV infection (median, +32 days after HSCT), timely blocking of CMV activation is important for high-risk patients after HID HSCT. Letermovir can attenuate clinically significant CMV infection without major toxicities in CMV-seropositive HSCT recipients (Marty et al., 2017). Our prediction model may help high-risk patients benefit from letermovir prophylaxis and spare low-risk patients from additional financially burdensome therapy, whose efficacy in different patient profiles should be confirmed in the future.

The reconstitution of CMV-specific CTLs is important for CMV prophylaxis after HID HSCT (Luo et al., 2010; Kato et al., 2015). In humanized CMV-infected mice, CTL therapy could promote the restoration of graft-derived endogenous CMV-specific immunity *in vivo* and combat systemic CMV infections (Zhao et al., 2020). In a registry clinical study (NCT02985775), Zhao et al. (2020) reported that CMV-specific CTL therapy was safe and well-tolerated. First-line therapy with CMV-CTLs promoted the quantitative and functional recovery of CTLs in high-risk patients (i.e., patients who developed aGVHD, followed by CMV reactivation), which was associated with CMV clearance. Thus, our predicted model may also help in the direct risk stratification of CMV-CTL therapy in HID HSCT recipients.

Donor/recipient CMV serological status could influence CMV infection after allo-HSCT; CMV-seronegative donors over seropositive donors for seropositive recipients have negative effects (Ljungman et al., 2019). However, this was not included in this model. This may be due to the fact that most of the cases were CMV donor positive/recipient positive in the present study. Fang et al. (2009) reported that the seroprevalence of CMV in the Chinese population was up to 97% in adults aged 20–25 years old. Therefore, it is difficult to further identify the association between CMV donor/recipient status and CMV reactivation after HID HSCT. In contrast, Crocchiolo et al. (2016) reported that in HID HSCT recipients receiving PTCy, the cumulative incidence of CMV infection was 45%, 52%, and 51% for donor-positive/recipient-negative, donor-positive/recipient-positive, and donor-negative/recipient-positive groups, respectively. It has been suggested that the influence of donor/recipient CMV serological status on CMV infection after HID HSCT may be relatively weak.

Many studies have reported that aGVHD is an important risk factor for refractory/recurrent CMV infections (Emery et al., 2013; Liu et al., 2015; Chemaly et al., 2019). However, some patients experienced CMV infection without aGVHD in clinical practice. In the present study, with the help of our model, we identified patients without aGVHD who would have a higher risk of refractory/recurrent CMV infections after HID HSCT.

Some studies have reported that CMV activation can help decrease the risk of posttransplant relapse (Green et al., 2013; Ito et al., 2013; Takenaka et al., 2015). However, other studies have not observed an association between CMV infection and relapse. In a large multicenter study enrolling nearly 9,500 patients, no impact of CMV (serology or infection) on relapse was found (Teira et al., 2016). In addition, Jeljeli et al. (2014) observed a higher risk of relapse with CMV infection. In the present study, low-risk patients showed a lower risk of relapse and better LFS, which suggested that CMV infection does not help decrease relapse in AL patients following HID HSCT.

According to the theory of machine learning, adding more variables increases the capacity and performance upper bound of the predictive model [Vapnik-Chervonenkis dimension (Abu-Mostafa, 1989; Blumer et al., 1989)] but also increases the complexity of the predictive model. Additionally, too many variables can make a model difficult to apply clinically. Thus, obligate variables seem to be a balanced approach, which can be the foundation of the model. Based on this, other variables can be further added to this model to construct the final model (Mitchell, 2006; Han et al., 2011). Age and gender are the most common obligate variables because they are easy to acquire in the real world and adding them usually does not increase the clinical burden (Nemati et al., 2018; Rajkomar et al., 2018; Zoabi et al., 2021). Hence, we extracted “age” and “female recipient” as the factors involved in predicting the development of refractory/recurrent CMV infection.

We observed that some variables that differed between the low- and high-risk groups were not included in the final model. This may be because the machine model was built for prediction, and its outcome was transformed through a sigmoid activation function to present the numeric probability between 0 and 1. In addition, the selection was realized using generalized linear models with binomial exponential family distribution of Statsmodels v0.13.0 statistical models module in Python 3.8 based on the Anaconda3 development platform.

The present study has some limitations. First, although we validated the model in the validation cohort, this was a single-center study and the sample size of the validation cohort was relatively small. Therefore, the model should be evaluated using independent cohorts in multicenter studies. Second, we observed that high-risk patients had a trend of a higher cumulative incidence of CMV infection compared to that of low-risk patients; however, only five patients experienced CMV infection, and we could not further identify the predicted value for CMV infection. Further research should assess the predictive power of our model. Third, ATG was administered to prevent GVHD in all HID HSCT recipients. Ninety-four percent of the conditioning regimens for HID HSCT contained ATG (Xu et al., 2021). Thus, the predicted value of our model should be further confirmed in patients receiving HID HSCT with PTCY for GVHD prophylaxis and in those receiving identical sibling donor HSCT. Lastly, most of the patients were in CR1 and had a low comorbidity burden (HCT-CI scores of 0) before HSCT in the present study. However, we also observed that our model could predict the occurrence of refractory/recurrent CMV infection in patients who had a higher comorbidity burden (HCT-CI scores ≥1) and those with >CR1 before HSCT. As our previous study showed that patients with high-risk HCT-CI scores had poorer OS and higher NRM after HID HSCT (Mo et al., 2013), we strengthened comorbidity screening before HSCT, and some patients with high comorbidity burdens did not receive HSCT after our previous study. Thus, the efficacy of our model should be further evaluated in patients with a high comorbidity burden and who were in non-remission before HSCT.

We have established a comprehensive model that can predict the development of refractory/recurrent CMV infection in HID HSCT recipients. It is a concise model that can be popularized easily and provides an appropriate 3-week window for risk stratification CMV prophylaxis in HID HSCT recipients. In the future, prospective multicenter studies can assess the efficacy of our prediction model further.

## Data Availability Statement

The raw data supporting the conclusions of this article will be made available by the authors without undue reservation.

## Ethics Statement

The studies involving human participants were reviewed and approved by the Institutional Review Board of Peking University People’s Hospital. Written informed consent to participate in this study was provided by the participants' legal guardian/next of kin.

## Author Contributions

X-DM and X-JH designed the study. JW, M-ZS, X-HZ, L-PX, YW, C-HY, HC, Y-HC, WH, F-RW, J-ZW, and K-YL conducted data collection. M-ZS, S-DH, X-DM, and X-JH conducted data analysis and drafted the article. All authors participated in interpreting the data, preparing the article, and approving the final version.

## Funding

This work was supported by the Program of the National Natural Science Foundation of China (grant number 82170208), the Foundation for Innovative Research Groups of the National Natural Science Foundation of China (grant number 81621001), the Key Program of the National Natural Science Foundation of China (grant number 81930004), CAMS Innovation Fund for Medical Sciences (CIFMS) (grant number 2019-I2M-5-034), and the Fundamental Research Funds for the Central Universities.

## Conflict of Interest

The authors declare that the research was conducted in the absence of any commercial or financial relationships that could be construed as a potential conflict of interest.

## Publisher’s Note

All claims expressed in this article are solely those of the authors and do not necessarily represent those of their affiliated organizations, or those of the publisher, the editors and the reviewers. Any product that may be evaluated in this article, or claim that may be made by its manufacturer, is not guaranteed or endorsed by the publisher.
