# Supplementary material for: A Predicted Model for Refractory/Recurrent Cytomegalovirus Infection in Acute Leukemia Patients After Haploidentical Hematopoietic Stem Cell Transplantation
Source: Front Cell Infect Microbiol. 2022 Mar 22;12:862526. doi: 10.3389/fcimb.2022.862526 (PMC8981086; doi:10.3389/fcimb.2022.862526)
Supplement: Supplementary file 1 [file DataSheet_1.docx]

Supplementary Appendix

**A predicted model for refractory/recurrent cytomegalovirus infection in acute leukemia patients after haploidentical hematopoietic stem cell transplantation**

**Contents**

**Supplementary methods**

Graft-versus-host disease (GVHD) prophylaxis protocol……………………………...2

Infection prophylaxis other than CMV………………………………………………...3

Definitions……………………………………………………………………………..4

Variables for building machine learning models………………………………………5

**Supplementary Tables**

Supplementary Table 1. Generalized Linear Model Regression Results……………...6

Supplementary Table 2. Table of confusion in the training cohort…………………….7

Supplementary Table 3. Table of confusion in the validation cohort………………….8

Supplementary Table 4. Patient characteristics in the low- and high-risk groups …….9

**Supplementary Figure**

Supplementary Figure 1. p-value iteration during backward feature selection ....……11

Supplementary Figure 2. The 100-day cumulative incidence of refractory/recurrent CMV infection in patients with HCT-CI scores of 0 between the low- and high-risk groups.....………………………………………………………………………..……12

Supplementary Figure 3. The 100-day cumulative incidence of refractory/recurrent CMV infection in patients with HCT-CI scores of ≥ 1 between the low- and high-risk groups.....………………………………………………………………………..……13

Supplementary Figure 4. The 100-day cumulative incidence of refractory/recurrent CMV infection in patients in CR1 between the low- and high-risk groups……….…. 14

Supplementary Figure 5. The 100-day cumulative incidence of refractory/recurrent CMV infection in patients in > CR1 between the low- and high-risk groups……...… 15

Supplementary Figure 6. The 100-day cumulative incidence of aGVHD after HID HSCT between the low- and high-risk groups…….………………………………..... 16

Supplementary Figure 7. The 1-year cumulative incidence of cGVHD after HID HSCT between the low- and high-risk groups………….…………………………………... 17

Supplementary Figure 8. The 1-year cumulative incidence of PGF after HID HSCT between the low- and high-risk groups……………………………………..………... 18

**Supplementary methods**

**Graft-versus-host disease (GVHD) prophylaxis protocol**

All the HID HSCT recipients received rabbit antithymocyte globulin (ATG, thymoglobulin, 2.5 mg/kg/day, days −5 to −2; Sanofi, France), cyclosporine A (CsA), mycophenolate mofetil (MMF), and short-term methotrexate (MTX) for GVHD prophylaxis (day 0 being the first day of donor cell infusion). CsA (2.5 mg/kg, q12h, intravenous [i.v.]) was used from day −3, of which the trough concentration was adjusted to 150–250 ng/mL. It was switched to oral administration when the patient’s bowel function returned to normal. From day −3, 0.25–0.5 g of MMF was administered orally every 12 h, then it was discontinued when neutrophil engraftment was achieved. Following graft infusion, a dose of 15 mg/m^2^ of MTX was administered i.v. on day +1, as well as a dose of 10 mg/m^2^ on days +3, +5, and +11. Particularly, patients with mother donors or collateral relative donors could receive two doses of 14.5 mg/kg cyclophosphamide on days +3 and +4 post-HSCT based on ATG (n=35).

**Infection prophylaxis other than CMV**

Patients are hospitalized in rooms with high-efficiency particulate arresting (HEPA) air filters for 3–4 weeks, that is, from day –10 until the time at which neutrophil engraftment was achieved. All patients receive antibiotics for gastrointestinal decontamination during period of conditioning regimen and neutropenia (i.e., fluoroquinolone is orally given to adults and injection liquid of gentamicin is orally given to children). Patients without an invasive fungal disease (IFD) before HSCT receive posaconazole from days –10 to +75 to prevent IFD, and those who have an IFD before HID HSCT should receive previously effective antifungal drug from days –9 to +100 to prevent the recurrence of IFD. Patients receive trimethoprim-sulfamethoxazole to prevent a *Pneumocystis jirovecii* infection from days –9 to +180.

**Definitions**

Disease risk index (DRI) was defined and graded according to the criteria of Armand et al. The neutrophil engraftment was defined as the first of 3 consecutive days that the absolute neutrophils achieved 0.5×10^9^/L without G-CSF, and platelet engraftment was defined as the first of 7 consecutive days that the absolute platelets achieved 20×10^9^/L with transfusion independence. PGF was defined as persistent neutropenia (≤ 0.5×10^9^ L^−1^), thrombocytopenia (platelets ≤ 20×10^9^ L^−1^), and/or hemoglobin ≤ 70 g L^−1^ for at least three consecutive days by day 28 post-transplantation, transfusion-dependence, associated with hypoplastic-aplastic bone marrow, and complete donor chimerism without concurrent GVHD or disease relapse. Relapse was defined as morphologic evidence of disease in peripheral blood, bone marrow, or extramedullary samples. Leukemia-free survival (LFS) was defined as the survival period with continuous complete remission after transplantation. Non-relapse mortality (NRM) was defined as death without relapse after transplantation. Overall survival (OS) was the period between the date of transplantation and death from any cause.

**Variables for building machine learning models**

Variables included age, gender, underlying disease, disease status before HSCT, disease risk index (DRI), hematopoietic cell transplantation-specific comorbidity index (HCTCI) score, human leukocyte antigen (HLA) disparity, cytomegalovirus (CMV) serostatus, conditioning regimen, the cumulative dose of prednisone during pre-engraftment phase, donor/recipient gender matched, donor/recipient relation, mononuclear cells (MNC), and CD34+ cell counts in the grafts.

**Variables for training machine learning models**

| Variables |  |
| --- | --- |
| Age (years) | actual numerical value |
| Gender | male=0; female=1 |
| Underlying disease | acute myeloid leukemia=0; acute lymphoblastic leukemia =1 |
| Disease status before HSCT | CR1=0; > CR1=1 |
| DRI | low risk=0; intermediate risk=1; high risk=2 |
| HCT-CI score | actual numerical value |
| HLA disparity | 1 locus=0; ≥2 loci=1 |
| CMV serostatus | D+/R-=0; D+/R+=1; D-/R+=2 |
| Conditioning regimen | TBI-based=0; Chemotherapy-based=1 |
| The cumulative dose of prednisone during pre-engraftment phase (mg/kg) | actual numerical value |
| Donor/recipient gender matched | others=0; female donor/male recipient=1 |
| Donor/recipient relation | immediate related donors, others=0; immediate related donors, mother donors=1; collateral related donors=2 |
| Mononuclear cell counts in graft (×10^8^/kg) | actual numerical value |
| CD34^+^ cell counts in graft (×10^6^/kg) | actual numerical value |

CMV, cytomegalovirus; CR, complete remission; D, donor; DRI, disease risk index; HCT-CI, hematopoietic cell transplantation-specific comorbidity index; HLA, human leukocyte antigen; HSCT, hematopoietic stem cell transplant; R, recipient; TBI, total body irradiation.

**Supplementary Table 1. Generalized Linear Model Regression Results**

| **Variables** | **Coef** | **Std err** | **Z** | ***P***  **value** | **95%CI** | |
| --- | --- | --- | --- | --- | --- | --- |
|  |  |  |  |  | **0.025** | **0.975** |
| Const | -1.2926 | 0.703 | -1.838 | 0.066 | -2.671 | 0.086 |
| CD34+ cells count in graft (×10^6^/kg) | -0.0771 | 0.067 | -1.157 | 0.247 | -0.208 | 0.054 |
| The cumulative dose of prednisone during pre-engraftment phase (mg/kg) | 0.0963 | 0.077 | 1.257 | 0.209 | -0.054 | 0.246 |
| Underlying disease | 0.5492 | 0.357 | 1.540 | 0.124 | -0.150 | 1.248 |
| Gender | -0.0696 | 0.376 | -0.185 | 0.853 | -0.808 | 0.668 |
| Age (years) | 0.0322 | 0.012 | 2.748 | 0.006 | 0.009 | 0.055 |
| CI, confidence interval; const, constant.  Underlying disease: acute myeloid leukemia: 0; acute lymphoblastic leukemia: 1;  Gender: male: 0; female: 1;  Age, CD34+ cells count in graft, the cumulative dose of prednisone during pre-engraftment phase: actual numerical value. | | | | | | |

**Supplementary Table 2. Table of confusion in the training cohort**

|  | Predicted refractory/resistant  CMV infection negative | Predicted refractory/resistant  CMV infection positive |
| --- | --- | --- |
| Refractory/resistant  CMV infection  negative | 66  (True negative) | 23  (False positive) |
| Refractory/resistant  CMV infection  positive | 38  (False negative) | 43  (True positive) |

**Supplementary Table 3. Table of confusion in the validation cohort**

|  | Predicted refractory/resistant  CMV infection negative | Predicted refractory/resistant  CMV infection positive |
| --- | --- | --- |
| Refractory/resistant  CMV infection  negative | 36  (True negative) | 18  (False positive) |
| Refractory/resistant  CMV infection  positive | 36  (False negative) | 29  (True positive) |

**Supplementary Table 4. Patient characteristics**

| **Characteristics** | **Low-risk**  **group**  **(*n*=176)** | **High-risk**  **group**  **(*n*=113)** | ***P* value** |
| --- | --- | --- | --- |
| Median age at allo-HSCT, years (range) | 18.5 (1–53) | 43 (8–66) | **<0.001** |
| Gender, *n* (%) |  |  | 0.690 |
| Male | 37 (21.0) | 26 (23.0) |  |
| Female | 139 (79.0) | 87 (77.0) |  |
| Underlying disease, *n* (%) |  |  | 0.101 |
| Acute myeloid leukemia | 103 (58.5) | 55 (48.7) |  |
| Acute lymphoblastic leukemia | 73 (41.5) | 58 (51.3) |  |
| Disease status before allo-HSCT, *n* (%) |  |  | 0.700 |
| CR1 | 132 (75.0) | 87 (77.0) |  |
| > CR1 | 44 (25.0) | 26 (23.0) |  |
| Disease risk index before allo-HSCT, *n* (%) |  |  | 0.545 |
| Low risk | 6 (3.4) | 7 (6.2) |  |
| Intermediate risk | 134 (76.1) | 84 (74.3) |  |
| High risk | 36 (20.5) | 22 (19.5) |  |
| HCT-CI scores before allo-HSCT, *n* (%) |  |  | **<0.001** |
| 0 (low-risk) | 152 (86.4) | 75 (66.4) |  |
| 1-2 (intermediate-risk) | 19 (10.8) | 31 (27.4) |  |
| ≥3 (high-risk) | 5 (2.8) | 7 (6.2) |  |
| Number of HLA-A, HLA-B, HLA-DR mismatches, *n* (%) |  |  | 0.770 |
| 1 locu | 4 (2.3) | 2 (1.8) |  |
| ≥ 2 loci | 172 (97.7) | 111 (98.2) |  |
| Cytomegalovirus serostatus before HSCT, *n* (%) |  |  | **0.002** |
| Donor+/recipient- | 9 (5.1) | 0 (0.0) |  |
| Donor+/recipient+ | 164 (93.2) | 106 (93.8) |  |
| Donor-/recipient+ | 3 (1.7) | 7 (6.2) |  |
| Conditioning regimen, *n* (%) |  |  | 0.561 |
| Chemotherapy-based regimen | 173 (98.3) | 112 (99.1) |  |
| TBI-based regimen | 3 (1.7) | 1 (0.9) |  |
| Median cumulative dose of prednisone during pre-engraftment phase, (mg/kg) | 3.53 (0.76–11.54) | 3.77 (0.72–16.94) | 0.189 |
| Donor/recipient gender matched, *n* (%) |  |  | 0.648 |
| Female donor/male recipient combination | 35 (19.9) | 25 (22.1) |  |
| Others | 141 (80.1) | 88 (77.9) |  |
| Donor/recipient relation, *n* (%) |  |  | 0.194 |
| Mother donor | 22 (12.5) | 7 (6.1) |  |
| Collateral donor | 3 (1.7) | 3 (2.7) |  |
| Others | 151 (85.8) | 103 (91.2) |  |
| MNC counts in graft, median (range, ×10^8^/kg) | 9.54 (5.20–27.52) | 8.90 (5.97–16.02) | **0.043** |
| CD34+ cell counts in graft, median (range, ×10^6^/kg) | 4.33 (0.75–29.35) | 2.76 (1.33-8.60) | **<0.001** |
| Median follow-up of survivors, days (range) | 277 (52–490) | 235.5 (66–409) | **0.005** |

allo-HSCT, allogeneic hematopoietic stem cell transplantation; CR, complete remission; HLA, human leukocyte antigen; HCTCI, hematopoietic cell transplantation-specific comorbidity index; MNC, mononuclear cells; TBI, total body irradiation.


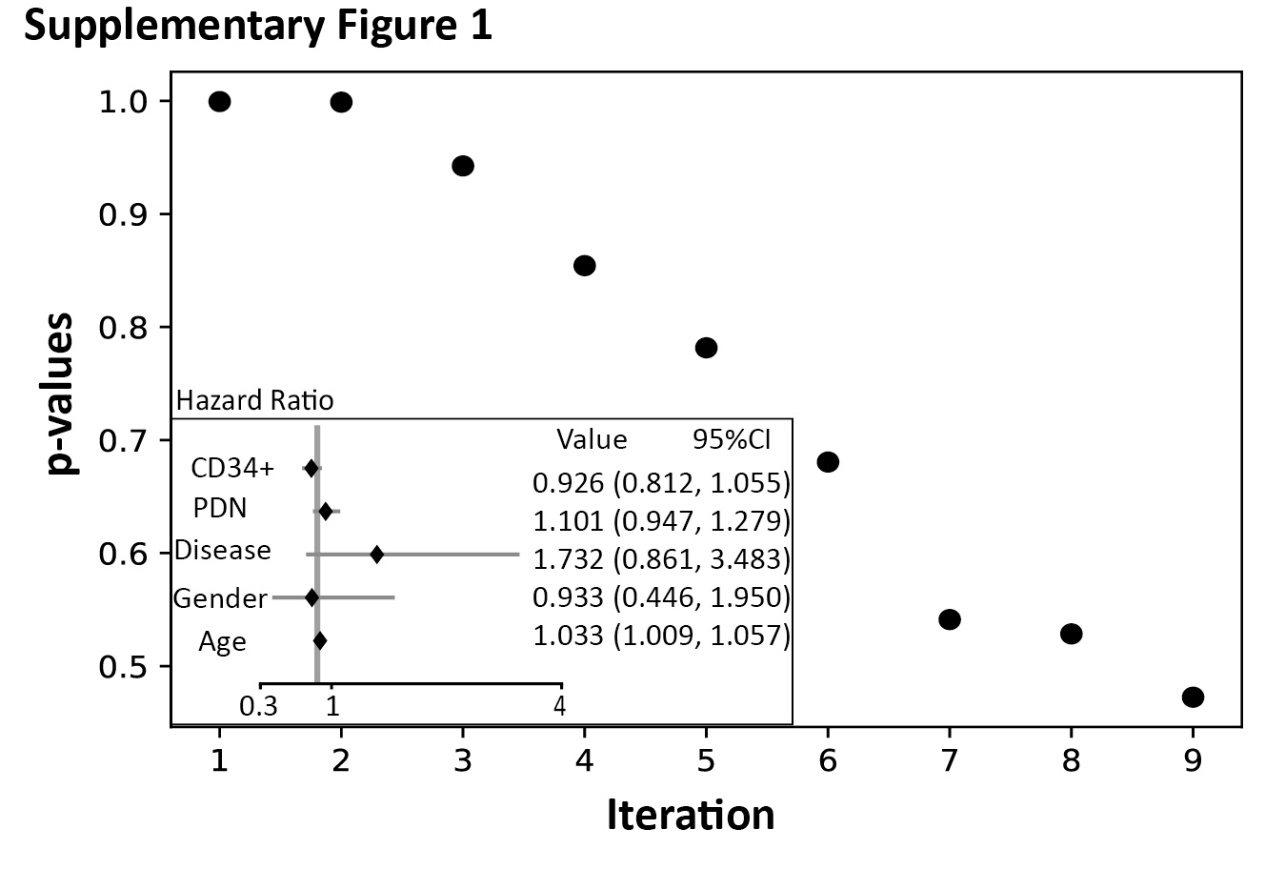


**Supplementary Figure 1. p-value iteration during backward feature selection.** CD34+, CD34+ cells count in graft (×10^6^/kg); PDN, the cumulative dose of prednisone during pre-engraftment phase (mg/kg); Disease, underlying disease.

**
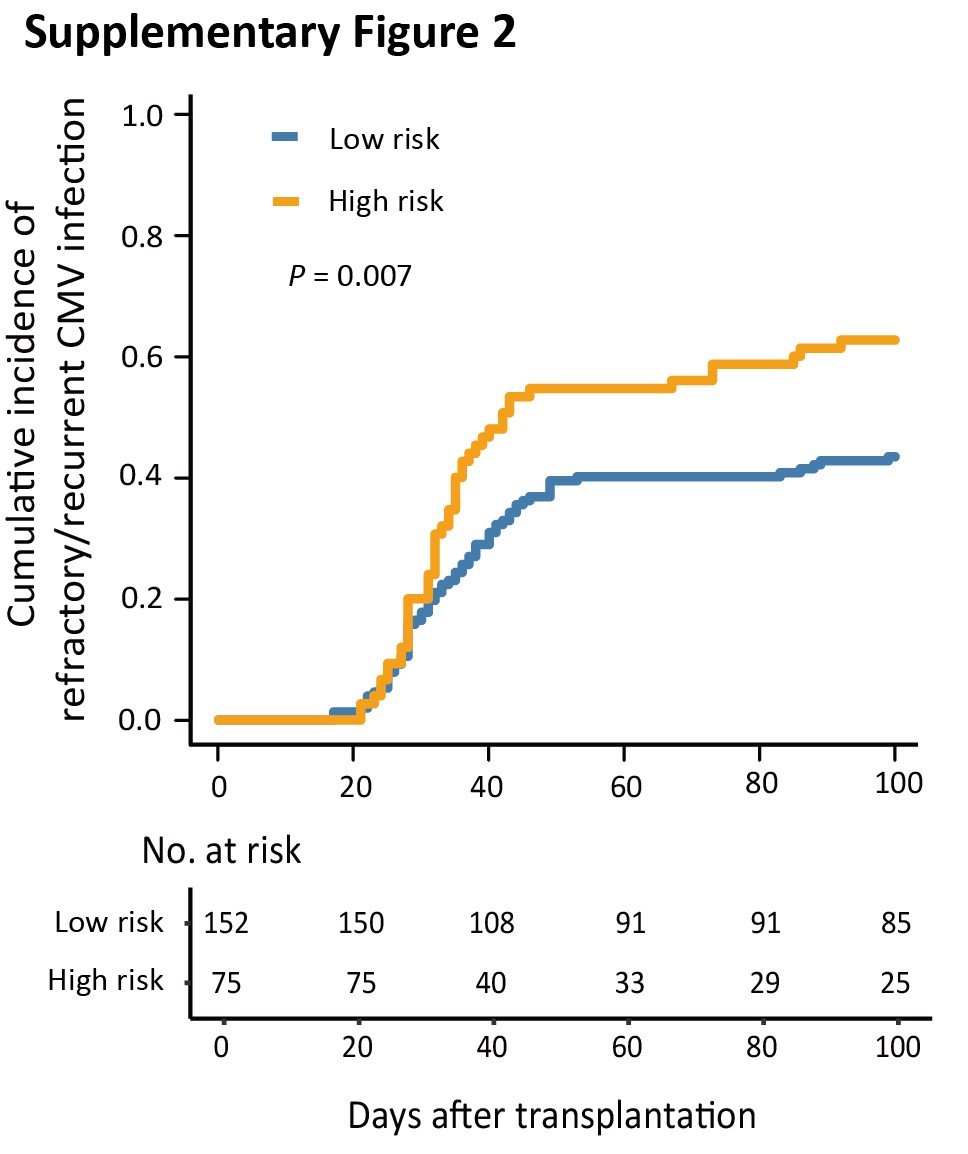
**

**Supplementary Figure 2. The 100-day cumulative incidence of refractory/recurrent CMV infection in patients with HCT-CI scores of 0 between the low- and high-risk groups.**

**
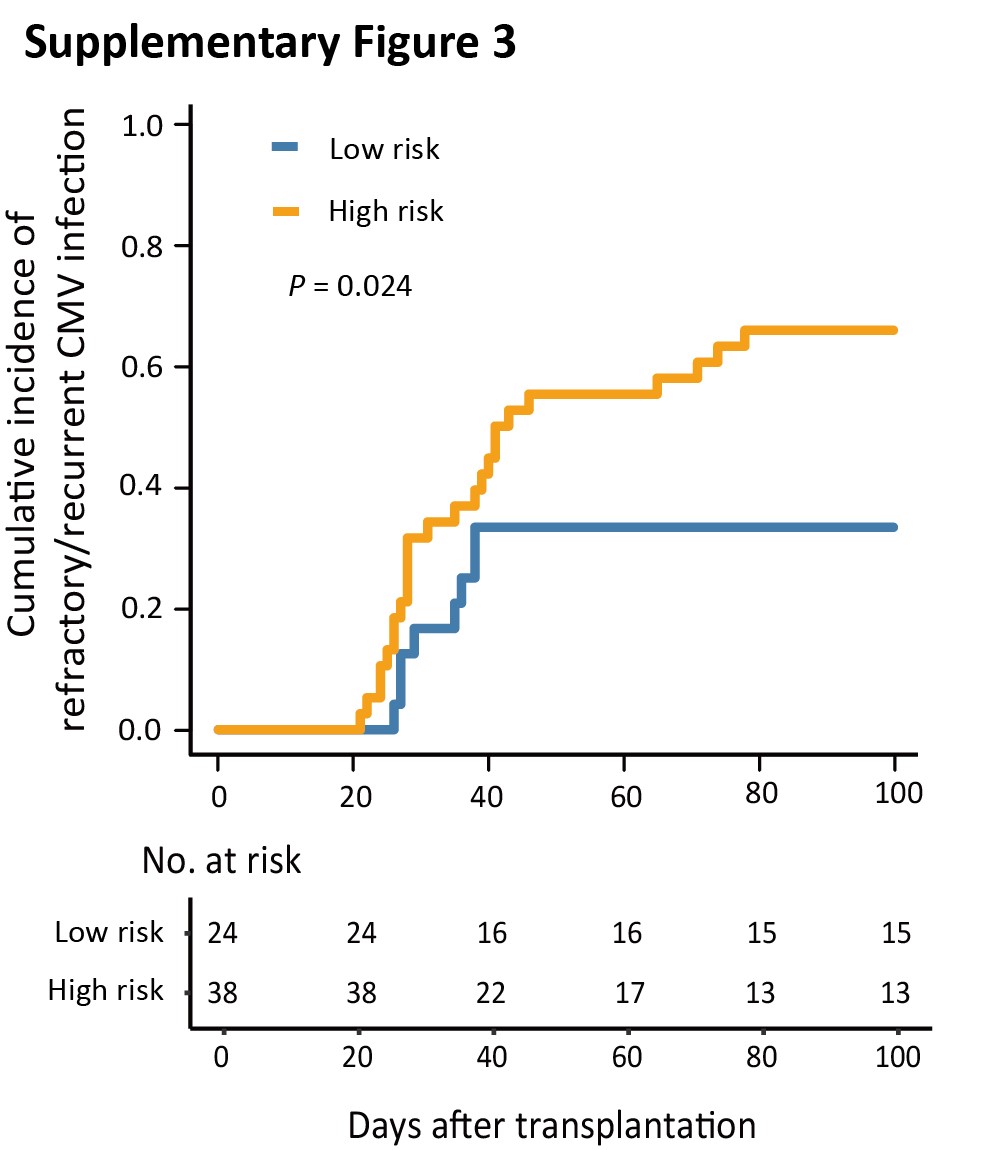
**

**Supplementary Figure 3. The 100-day cumulative incidence of refractory/recurrent CMV infection in patients with HCT-CI scores of ≥ 1 between the low- and high-risk groups.**

**
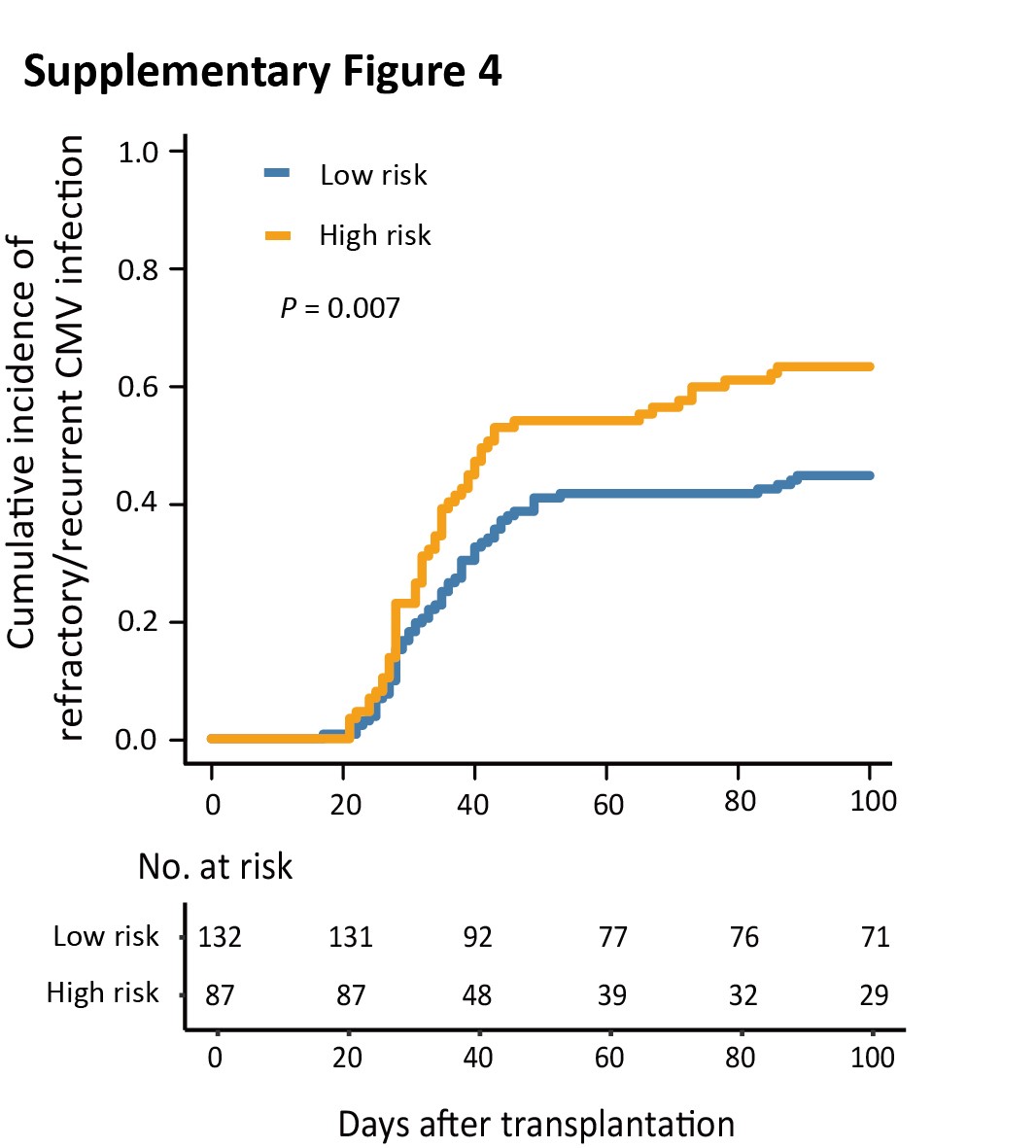
**

**Supplementary Figure 4. The 100-day cumulative incidence of refractory/recurrent CMV infection in patients in** **CR1 between the low- and high-risk groups.**

**
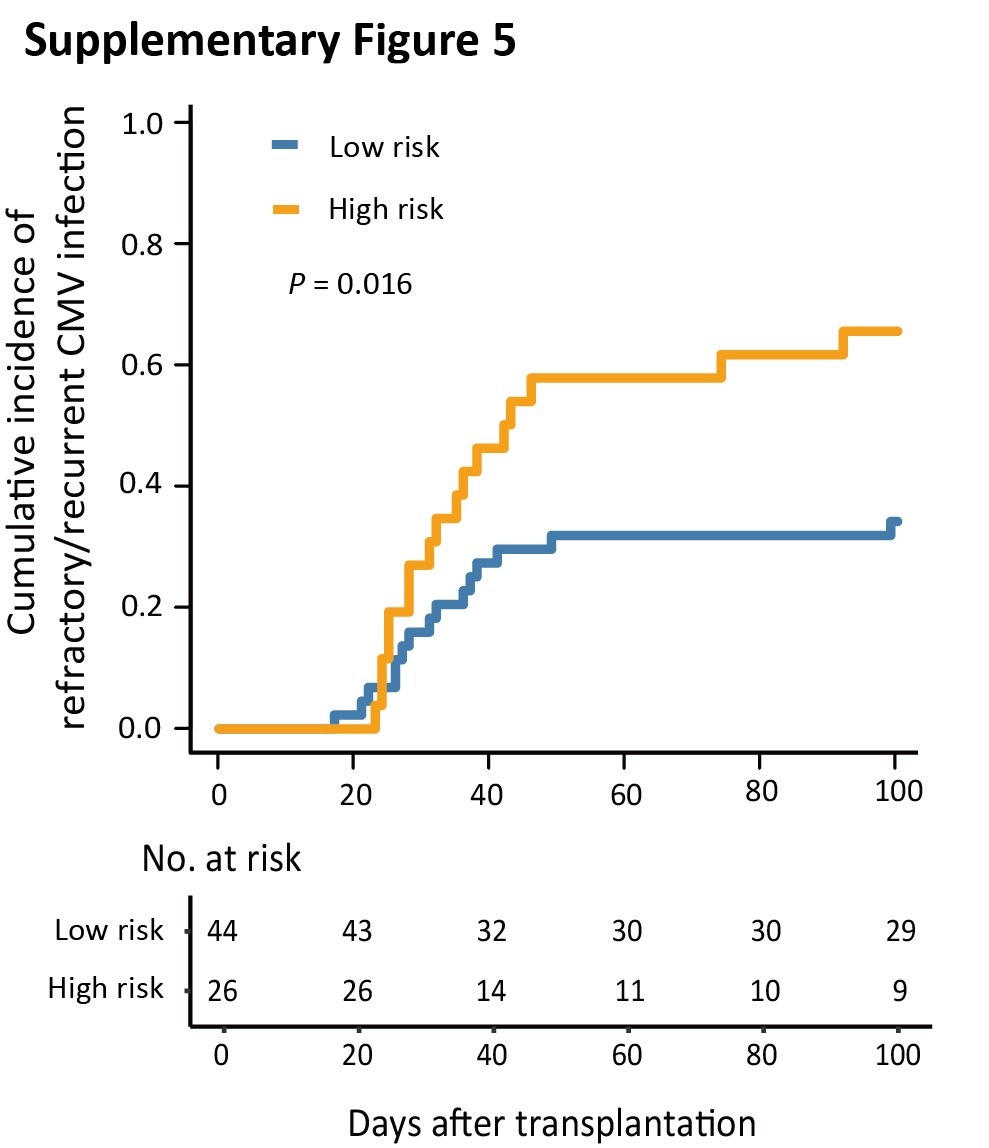
**

**Supplementary Figure 5. The 100-day cumulative incidence of refractory/recurrent CMV infection in patients in** **> CR1 between the low- and high-risk groups.**

**
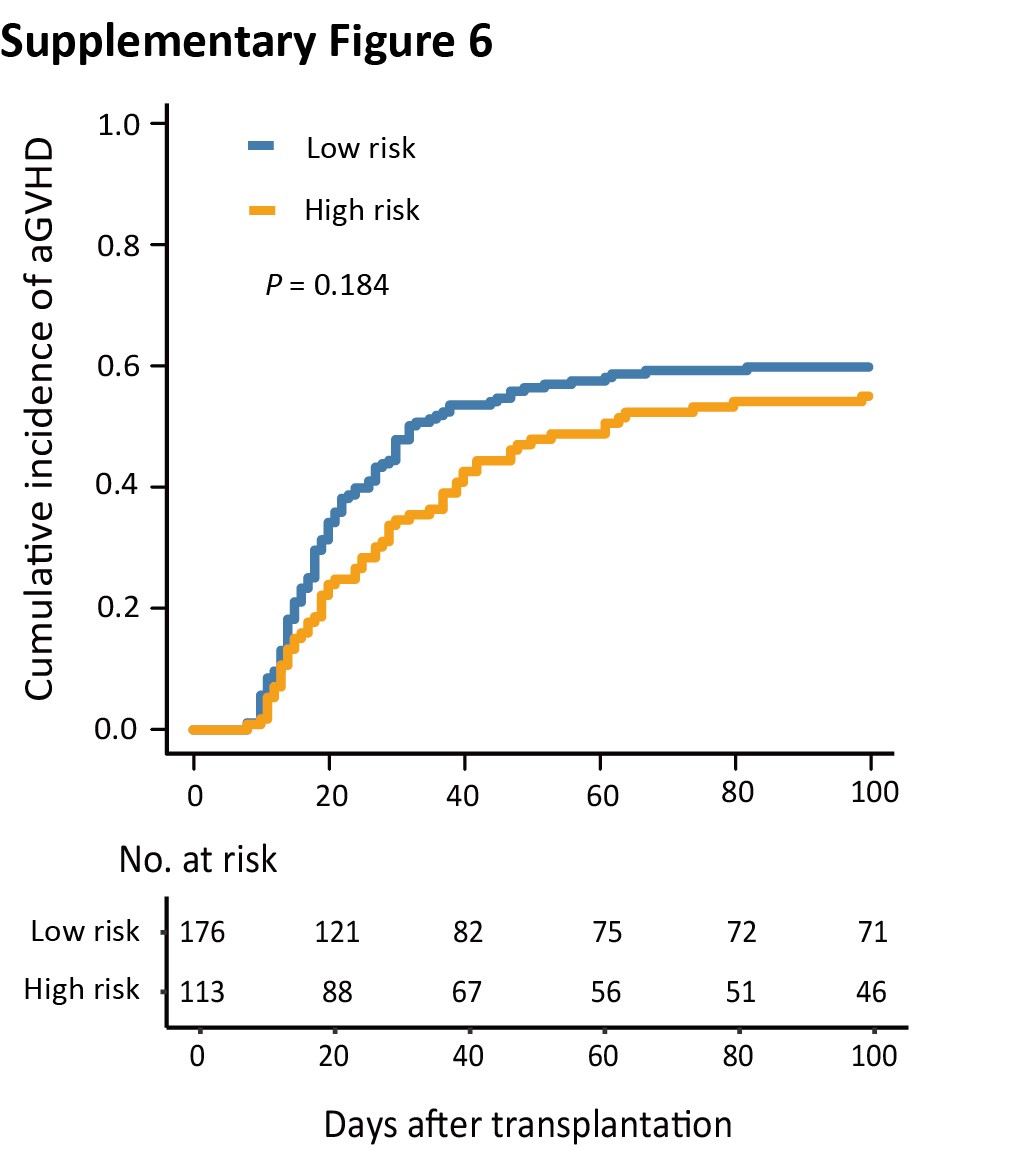
**

**Supplementary Figure 6. The 100-day cumulative incidence of aGVHD after HID HSCT between the low- and high-risk groups.**

**
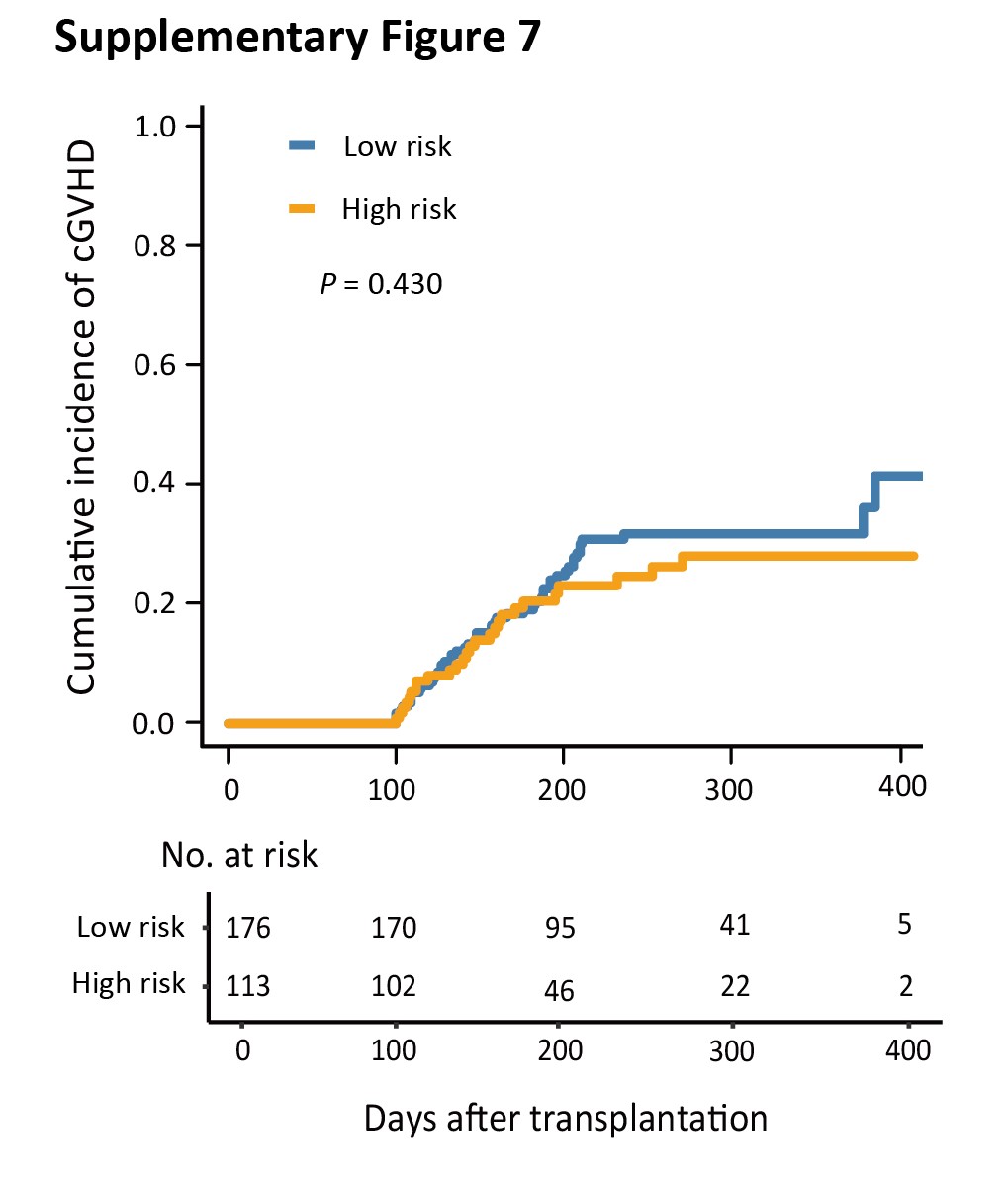
**

**Supplementary Figure 7. The 1-year cumulative incidence of cGVHD after HID HSCT between the low- and high-risk groups.**

**
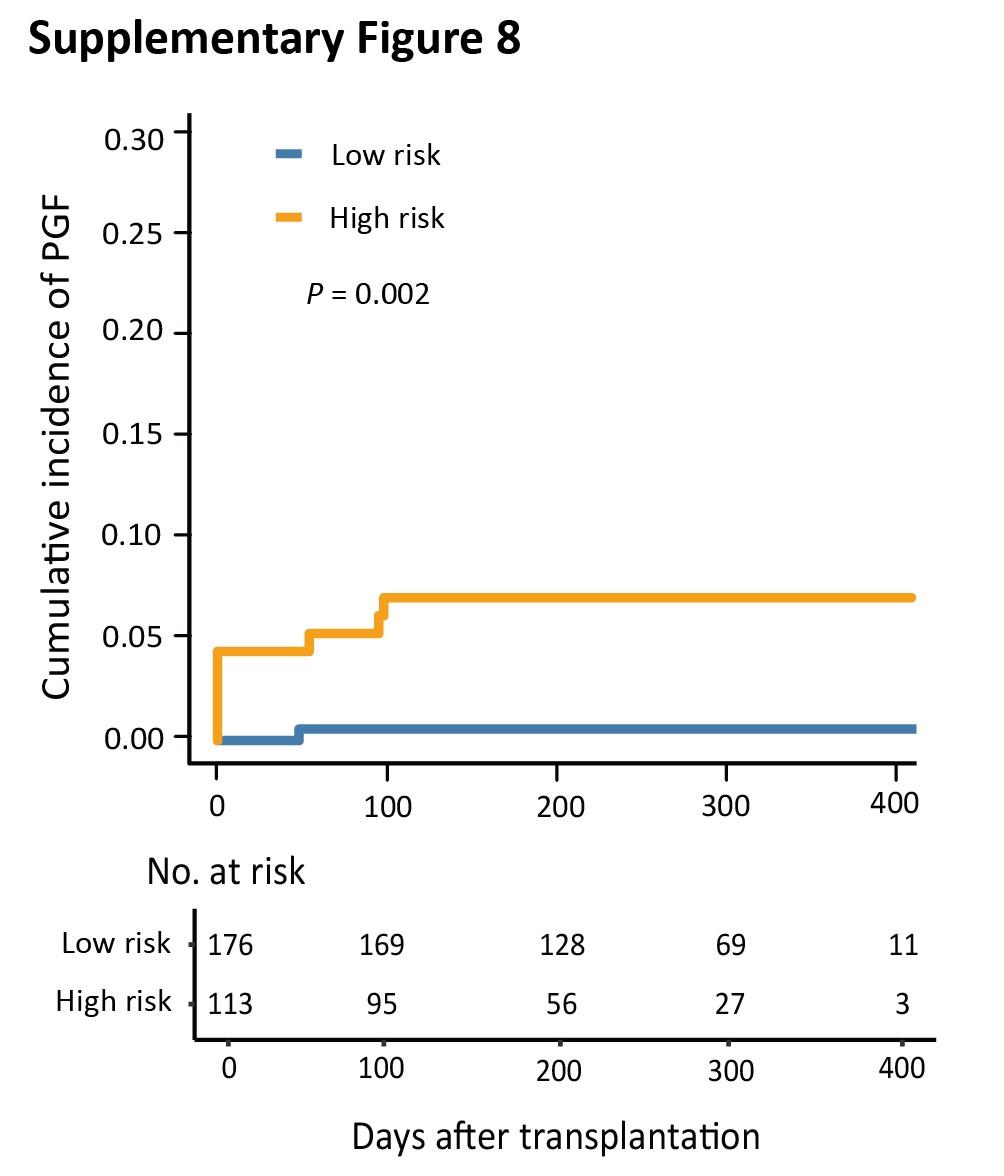
**

**Supplementary Figure 8. The 1-year cumulative incidence of PGF after HID HSCT between the low- and high-risk groups.**
